# Severe hypothyroidism as a trigger for Brugada-type ECG abnormalities: a case report and literature review

**DOI:** 10.20945/2359-4292-2023-0027

**Published:** 2024-02-08

**Authors:** Fabio Bioletto, Daniela Cuboni, Emanuele Varaldo, Chiara Bona, Alessandro Maria Berton, Mauro Maccario, Nunzia Prencipe

**Affiliations:** 1 Università di Torino Dipartimento di Scienze Mediche Divisione di Endocrinologia, Diabetologia e Metabolismo Torino Italia Divisione di Endocrinologia, Diabetologia e Metabolismo, Dipartimento di Scienze Mediche, Università di Torino, Torino, Italia

## Abstract

Brugada syndrome (BrS) is an inherited disorder that can cause ventricular fibrillation and sudden cardiac death in individuals with otherwise structurally normal hearts. Several provoking factors are known to potentially unmask or exacerbate a typical Brugada ECG pattern in predisposed subjects. Hypothyroidism has been suggested as one of these triggers, but the exact mechanisms underlying this relationship remain poorly understood. Moreover, the severity of thyroid dysfunction beyond which a Brugada-type ECG alteration might be triggered is still unclear. We report the case of a 33-year-old male who displayed a Brugada type 1 ECG pattern and was diagnosed with severe hypothyroidism (TSH > 100 mU/L with undetectable levels of fT4 and fT3). Hormonal replacement therapy with levothyroxine was initiated at increasing doses; serial biochemical and ECG controls were performed, initially every 3 weeks up to 15 weeks and afterward every 3 months. The regression of typical Brugada ECG waveforms could be seen at an early stage, when the patient was still taking a low dose of levothyroxine (37.5 µg/day, i.e., one-fourth of his final requirements of 150 µg/day), and laboratory tests still showed a marked alteration of thyroid hormonal parameters. Hypothyroidism may act as a trigger for Brugada-type ECG abnormalities, but a very severe alteration of the hormonal parameters is necessary to prompt these alterations. In our case, the initiation of replacement therapy with levothyroxine rapidly reversed the ECG modifications, even at a low subtherapeutic dose.

## INTRODUCTION

Brugada syndrome (BrS) is an autosomal dominant genetic disorder with variable penetrance that is characterized by abnormal ECG findings in conjunction with an increased risk of ventricular tachyarrhythmias and sudden cardiac death (1). Its prevalence is estimated to be approximately 0.5 cases per 1000 people, but with remarkable differences between ethnicities (2). The highest prevalence is reported in people living in South-Eastern Asia, where BrS is nine times more common than that in Caucasians (2). Moreover, it is characterized by a male predominance, as it is 5-10 times more frequent in males than in females (2,3).

The ECG findings consist of a pseudo-right bundle branch block together with an ST segment elevation in leads V1-V2, which appear more prominent when placing the electrodes on the 2^nd^ intercostal space (1,2,4). Two distinct patterns of ECG abnormalities are recognized (1,2,4):

Type 1 ("coved type"): high take-off ≥2 mm with respect to the isoelectric line; concave/rectilinear descending ST segment; negative symmetric T wave.

Type 2 ("saddle-back type"): high take-off ≥2 mm with respect to the isoelectric line; convex ST segment elevation with minimum ascent ≥0.5 mm and saddle-back morphology; positive T wave in V2, while of variable morphology in V1.

Only the first alteration is diagnostic for BrS, while the second one is only suggestive for it (1). The 2013 HRS/EHRA/APHRS Expert Consensus Statement (5) established that "BrS is definitively diagnosed when a type 1 ST-segment elevation is observed either spontaneously or after intravenous administration of a sodium channel blocking agent (ajmaline, flecainide, pilsicainide, or procainamide) in at least one right precordial lead (V1-V2), placed in a standard or a superior position (up to the 2^nd^ intercostal space)".

Although considered a genetic disease, a causative mutation is found only in approximately 30% of BrS patients, with the majority of pathogenic variants being located in the *SCN5A* gene (6,7). Several provoking factors are known to potentially unmask or exacerbate a typical Brugada ECG pattern in predisposed subjects (1,3), such as imbalances in the autonomic tone, fever, and various types of drugs. In addition, other conditions have been reported that may transiently affect ion channel function, potentially leading to Brugada-like ECG findings even in the absence of congenital dysfunction. These conditions have been termed "Brugada phenocopies" and may be induced by various mechanisms, including ischemia, myocardial/pericardial diseases, extracardiac mechanical compression, and metabolic alterations (8,9).

Severe hypothyroidism has been suggested as a potential trigger for Brugada-type ECG abnormalities in some patients of Asian descent (10–12); however, no data are available about its possible role as a trigger in patients with a different genetic background. Moreover, the severity of thyroid dysfunction beyond which a Brugada-type ECG alteration might be triggered is still unclear.

## LITERATURE REVIEW

The PubMed/Medline database was screened to identify relevant studies or reports about the association between Brugada pattern ECG abnormalities and hypothyroidism. The search algorithm used was based on the following search string: ("hypothyroidism") AND ("Brugada"). The search was updated through January 15^th,^ 2023. The search identified 9 results, but only 3 of them were actually pertinent to the topic of interest (10–12).

All 3 relevant results were case reports described in male patients of Asian descent (two Japanese patients, one Chinese patient). The age at presentation was heterogeneous, ranging from 46 years old in the case described by Zhao and cols. (12) to 77 years old in the case described by Kitahara and cols. (10). At physical examination, all patients showed clear signs of hypothyroidism, such as deep voice, myxedematous face and legs, and bradycardia. Thyroid function tests confirmed this clinical suspicion and demonstrated the presence of severe hypothyroidism, with TSH values > 100 mU/L and fT4 values below the lower limit of normality in all cases.

From a cardiological point of view, the ECG taken at rest showed a coved-type Brugada waveform in V1-V2 in two cases (10,11); in the third case, a coved-type waveform in V1 and a saddle-back waveform in V2 were present (12). None of the patients had a positive family history for sudden death, syncope, or palpitations; moreover, their pharmacological anamnesis was negative for drugs known to potentially induce Brugada-like ECG abnormalities. In all cases, the observed Brugada-type ECG alterations disappeared following the initiation of levothyroxine replacement therapy and the normalization of thyroid function tests. A genetic analysis for BrS was performed by Taira and cols. (11); this analysis demonstrated three common variants in the *SCN5A* gene (c.5963 T>G, c.1673 A>G, and c.3578 G>A), which are identified in more than 1% of normal subjects.

The most relevant characteristics of these three cases are presented in Table 1.

**Table 1:** Summary of available case reports in the literature

	Case 1 – Kitahara and cols. (10)	Case 2 – Taira and cols. (11)	Case 3 – Zhao and cols. (12)
Country	Japan	Japan	China
Patient’s characteristics	77-year-old male	52-year-old male	46-year-old male
Reason for hospital visit	Increase in transaminases	Diarrhea and cold sweating	Fatigue
Physical examination	– Deep voice – Myxedematous legs	– Deep voice – Myxedematous face and legs – Aurantiasis cutis in the hands	– Deep voice – Myxedematous face and legs – Aurantiasis cutis – Hertoghe sign
General laboratory data	↑ AST, ALT ↑ CK ↑ LDH	↔ AST, ALT ↑ CK ↑ LDH ↑ Total cholesterol	↑ AST, ALT ↑ CK ↑ LDH ↑ Total cholesterol
Thyroid function tests	TSH 145.8 mU/L fT4 0.5 ng/L TgAb + TPOAb +	TSH > 100 mU/L fT4 5 ng/L TgAb +	TSH > 100 mU/L fT4 < 1.5 ng/L TgAb + TPOAb +
Heart rate	61 bpm	46 bpm	58 bpm
Type of Brugada pattern at ECG	Coved type in V1-V2	Coved type in V1-V2	Coved type in V1, saddle-back type in V2
Genetic testing	N/A	SCN5A gene: c.5963 T>G c.1673 A>G c.3578 G>A	N/A
Disappearance of ECG alterations with the normalization of thyroid function tests	Yes	Yes	Yes

Abbreviations: ALT, alanine transaminase; AST, aspartate transaminase; bpm, beats per minute; CK, creatin kinase; ECG, electrocardiogram; fT4, free thyroxine; LDH, lactate dehydrogenase; N/A, not available; TgAb, anti-thyroglobulin antibodies; TPOAb, anti-thyroperoxidase antibodies; TSH, thyroid stimulating hormone.

## CASE PRESENTATION

We report the case of a 33-year-old male who was referred to our hospital in June 2021 due to worsening fatigue, slowing of movements and speech, weight gain, puffy facies, tongue enlargement and nonpitting edema. No significant comorbidities were reported by the patient in his past medical history; no chronic use of drugs was reported.

Through a biochemical assessment, a diagnosis of severe primary hypothyroidism was established. TSH was considerably elevated (>100 mU/L), and fT4 and fT3 were undetectable (<4.2 ng/L and <1.5 ng/L, respectively). Antithyroid peroxidase antibodies were markedly positive (>2.000 kIU/L). Through a thyroid ultrasound, the typical ultrasonographic features of chronic autoimmune thyroiditis could be observed. Other laboratory tests revealed significant myopathy (CK 3057 IU/L), a modest elevation of transaminases (AST 106 IU/L, ALT 95 IU/L) and a slight increase in serum creatinine (1.29 mg/dL), without electrolyte abnormalities.

**Figure 1:**
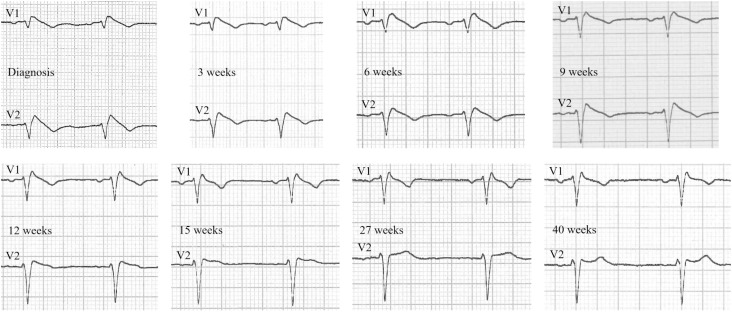
Progressive changes in ECG waveforms in V1-V2 (2^nd^ intercostal space) over time.

On standard ECG, performed as of routine practice upon admission to our hospital, Brugada-like alterations were noted; when repeating the ECG after moving V1-V2 leads to the 2^nd^ intercostal space, a typical Brugada type 1 pattern was evident (Figure 1). The patient reported no family history of Brugada syndrome or sudden cardiac death and no personal history of syncope. All his previous ECGs were normal (the last one performed two years earlier), as were those of his parents.

Following these diagnostic assessments, hormonal replacement therapy with levothyroxine was initiated, starting with a low dosage (12.5 µg/day) with a subsequent progressive increase, as displayed in Table 2. Moreover, the patient was advised to avoid any drug known to be a potential trigger of malignant arrhythmias in Brugada syndrome and to promptly treat any episode of hyperpyrexia with acetaminophen.

**Table 2:** Summary of clinical, biochemical, and electrocardiographic patient features over time. For laboratory data, the normal range is reported in square brackets

	Diagnosis	3 weeks	6 weeks	9 weeks	12 weeks	15 weeks	27 weeks	40 weeks
Levothyroxine dosage (μg/day)	0	12.5	25	37.5	50	75	100	150
Body weight (kg)	85.0	85.5	84.5	86.0	85.0	85.0	84.0	84.5
Levothyroxine dosage by body weight (μg/kg/day)	0	0.15	0.30	0.44	0.59	0.88	1.19	1.78
TSH (mU/L) [0.35-4.94]	>100.0	>100.0	>100.0	>100.0	84.2	87.3	42.2	0.8
fT4 (ng/L) [7.0-14.8]	<4.2	<4.2	<4.2	<4.2	4.9	5.9	6.0	9.0
fT3 (ng/L) [1.6-3.9]	<1.5	<1.5	<1.5	<1.5	1.6	2.0	2.1	3.0
TPOAb (kIU/L) [<10]	>2000	–	–	–	–	–	–	–
CK (IU/L) [<171]	3057	3448	–	–	477	–	226	–
AST (IU/L) [<50]	106	–	–	–	–	–	25	–
ALT (IU/L) [<50]	95	–	–	–	–	–	25	–
Na (mmol/L) [136-146]	137	136	–	–	141	–	–	139
K (mmol/L) [3.5-5.1]	3.5	3.5	–	–	4.0	–	–	3.8
Creatinine (mg/dL) [0.72-1.18]	1.29	1.16	–	–	1.04	–	–	0.78
Coved-type Brugada	Yes	Yes	Yes	No	No	No	No	No
ECG waveforms (V1-V2 in 2^nd^ intercostal space)								

Abbreviations: ALT, alanine transaminase; AST, aspartate transaminase; CK, creatine kinase; ECG, electrocardiogram; fT3, free triiodothyronine; fT4, free thyroxine; K, potassium; Na, sodium; TPOAb, anti-thyroperoxidase antibodies; TSH, thyroid-stimulating hormone.

Serial biochemical and ECG analyses were performed, initially every 3 weeks up to 15 weeks and then every 3 months. From a biochemical point of view, a progressive normalization of thyroid hormone examinations could be observed; a euthyroid state was finally obtained with a levothyroxine dosage of 150 µg/day, corresponding to 1.78 µg/kg/day when indexed for body weight (Table 2). All other biochemical alterations also resolved upon reaching euthyroidism (Table 2).

From a cardiological point of view, the acquisition of serial ECGs demonstrated a progressive normalization of the ECG pattern, with the regression of any type of Brugada-like alteration (Figure 1). Notably, the regression of Brugada waveforms could be seen at an early stage, when the patient was still taking very low doses of levothyroxine replacement therapy. More specifically, the diagnostic criteria for a type 1 Brugada pattern were no longer fulfilled starting from the 9^th^ week from the initiation of levothyroxine. At that point, indeed, the ECG still displayed a slightly enlarged r’ wave in V1-V2, but the ST segment morphology, although descending, was no longer of the concave type (i.e., concave/rectilinear) (Figure 1). This result was achieved when the patient was taking 37.5 µg/day of levothyroxine (i.e., one-fourth of his final requirements of 150 µg/day), and laboratory tests still showed a severe alteration of thyroid hormonal parameters (with a TSH > 100 mU/L and a fT4 still below the lower limit of detection of our laboratory) (Table 2). Three weeks later, in addition to the standard ECG, 12-lead Holter ECG monitoring was performed and did not detect any Brugada-type alterations throughout the entire recording time. Further modifications to the ECG waveforms could be seen at the subsequent medical visits, but a Brugada-like pattern never recurred (Figure 1).

## DISCUSSION

We presented the case of a patient with a Brugada type 1 ECG pattern triggered by a condition of severe hypothyroidism. This peculiar association had been previously described in three other patients in the literature, and all of these patients were of Asian origin (10–12). Notably, this is the first case describing such a situation in a Caucasian patient. This is an interesting point that shows that hypothyroidism might act as a trigger of a Brugada-type ECG also in patients with a different genetic background.

Although BrS is considered a genetic disease, a causative mutation is found in only approximately 30% of BrS patients (6,7). Moreover, the exact pathways by which the predisposing mutations interact with other factors to determine the final phenotype remain largely unclear (1,3,13). For example, BrS shows age- and sex-related penetrance, but the exact mechanisms remain poorly understood (1). The same applies to the triggers known to exacerbate this arrhythmia, whose specific action on myocardial cells is – in most of them – only partially elucidated. Within such a complex pathophysiological context, the demonstration that hypothyroidism might act as a trigger of a Brugada type 1 ECG pattern in a patient of non-Asian descent adds a further piece of evidence toward the understanding of this disease.

From a pathophysiological point of view, the interaction between thyroid function and myocardial ion channels remains unclear. Overall, the cause of Brugada-type ECG alterations is regarded to be a decrease in sodium inflow currents (I_Na_), with a relative increase in the transient outward current (I_to_). Although no clear data are available in humans, some physiological clues might be derived from animal studies. The evaluation of myocardial cells in rats suggested a positive relationship between thyroid hormone levels and the magnitude of I_Na_ (14,15). However, there is less concordance about the connection between thyroid function and I_to_. The main regulators of this current are represented by the potassium channel subunits K_V_4.2, K_V_4.3 and K_V_1.4, and different studies conducted on myocardial cells of rats found that their expression and function could be either unaffected (16,17), positively correlated (16,18,19), or negatively correlated (16) with thyroid hormone levels.

In our case, as in the others, the ECG alterations resolved after the initiation of replacement therapy with levothyroxine. An important strength of our report, however, is represented by the choice to follow-up the patient in a systematic and timely manner, with a biochemical assessment and ECG monitoring every 3 weeks up to the 15^th^ week and every 3 months thereafter. Such a systematic approach distinguishes our report from the previous ones and allowed us to more precisely correlate the ECG alterations with biochemical data. In this regard, our findings suggest that – even if hypothyroidism represents a triggering condition that could promote the appearance of a Brugada type 1 pattern on ECG – this is likely to happen only in the most severe forms of thyroid dysfunction, with very high values of TSH and undetectable serum levels of free thyroid hormones. As previously noted, in fact, in our case, the ECG waveforms did not fulfill the diagnostic criteria for a type 1 Brugada pattern at a very early stage, when the patient was still taking a low dose of levothyroxine (37.5 µg/day, i.e., one-fourth of the dose that was finally required) and was still displaying a marked alteration of the hormonal parameters (TSH > 100 mU/L, fT4 below the lower limit of detection).

In conclusion, our case shows that hypothyroidism may act as a trigger for Brugada-type ECG abnormalities. For the first time, this association was observed in a patient of non-Asian origin, demonstrating that it may also occur in patients with a different genetic background. Nevertheless, a very severe alteration of the hormonal parameters was necessary to prompt these alterations; the initiation of replacement therapy with levothyroxine rapidly reversed the ECG modifications, even at a low subtherapeutic dose.
